# Flavonoids with M_1_ Muscarinic Acetylcholine Receptor Binding Activity

**DOI:** 10.3390/molecules19078933

**Published:** 2014-06-27

**Authors:** Meyyammai Swaminathan, Chin Fei Chee, Sek Peng Chin, Michael J. C. Buckle, Noorsaadah Abd. Rahman, Stephen W. Doughty, Lip Yong Chung

**Affiliations:** 1 Department of Pharmacy, Faculty of Medicine, University of Malaya, 50603 Kuala Lumpur, Malaysia; E-Mails: oosh_mey@yahoo.com (M.S.); xueping.chin@gmail.com (S.P.C.); 2 Department of Chemistry, Faculty of Science, University of Malaya, 50603 Kuala Lumpur, Malaysia; E-Mails: cfchee@yahoo.com (C.F.C.); noorsaadah@um.edu.my (N.A.R.); 3 School of Pharmacy and Health Sciences, International Medical University, Jalan Jalil Perkasa 19, Bukit Jalil, 57000 Kuala Lumpur, Malaysia; 4 School of Pharmacy, University of Nottingham Malaysia Campus, Jalan Broga, 43500 Semenyih, Selangor Darul Ehsan, Malaysia; E-Mail: stephen.doughty@nottingham.edu.my

**Keywords:** Alzheimer’s disease, muscarinic acetylcholine receptor, binding activity, flavonoids, molecular modelling

## Abstract

Muscarinic acetylcholine receptor-active compounds have potential for the treatment of Alzheimer’s disease. In this study, a series of natural and synthetic flavones and flavonols was assayed *in vitro* for their ability to inhibit radioligand binding at human cloned M_1_ muscarinic receptors. Several compounds were found to possess competitive binding affinity (*K*_i_ = 40–110 µM), comparable to that of acetylcholine (*K*_i_ = 59 µM). Despite the fact that these compounds lack a positively-charged ammonium group under physiological conditions, molecular modelling studies suggested that they bind to the orthosteric site of the receptor, mainly through non-polar interactions.

## 1. Introduction

Muscarinic acetylcholine receptor (mAChR)-active compounds have promising potential for the treatment of Alzheimer’s disease (AD) [1]. The main pathological hallmarks of AD are cholinergic system dysfunction and the formation of extracellular neuritic plaques containing the β-amyloid peptide and neurofibrillary tangles composed of hyperphosphorylated forms of the tau protein. M_1_ mAChR agonists would therefore be expected not only to give symptomatic relief by improving cognitive functions but also to modify the progression of AD through the promotion of the non-amyloidogenic processing of amyloid precursor protein and inhibition of tau hyperphosphorylation [2,3].

Flavonoids are polyphenolic compounds which are commonly found in vegetables, fruits and drinks [4] and their human dietary intake is of the order of 100 mg/day [5]. They are known to possess a wide range of pharmacological properties, including neuroprotective effects and antiamyloidogenic activity [6,7,8,9]. There is also evidence from epidemiological studies that a higher dietary intake of flavonoids reduces the risk of AD and cognitive decline [10,11].

**Figure 1:**
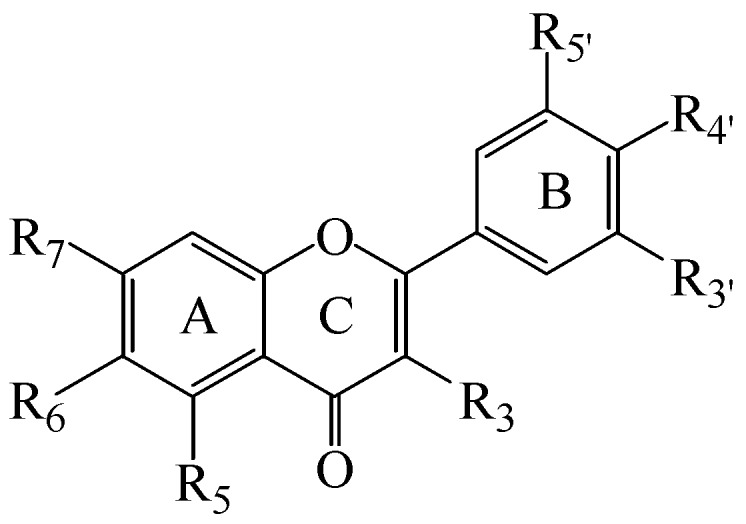
General structure of the investigated flavones and flavonols.

Most mAChR active ligands contain a positively-charged ammonium group under physiological conditions, but the fact that there are examples of active compounds that lack this feature [12,13,14,15] suggests that this may not be an absolute requirement. In our laboratory, we have previously identified a group of six polyoxygenated flavones from the bark extract of *Melicope subunifoliolata* (Stapf) T.G. Hartley which show competitive binding activity at muscarinic acetylcholine receptors [16]. In the current study, we have assayed a series of 13 structurally-related, natural and synthetic flavones and flavonols, with varying degrees of hydroxyl and methoxyl substitution at positions 5–7, 3'–5' and 3 in the A, B and C rings, respectively (Figure 1), in order to investigate whether this property might be a general characteristic of such compounds and have performed molecular modelling studies to explore further which structural features may be beneficial.

## 2. Results and Discussion

### 2.1. Synthesis of Flavones and Flavonols

**Scheme 1:**
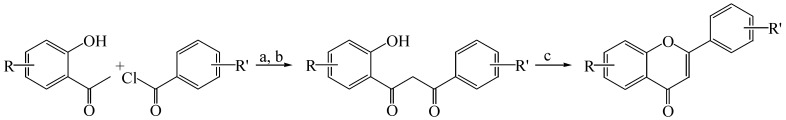
Synthesis of flavones **1**–**4**.

**Scheme 2:**
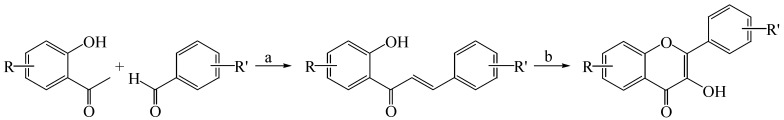
Synthesis of flavonols **9**–**10**.

Flavones **1**–**4** were prepared from the corresponding 2'-hydroxyacetophenones and benzoyl chlorides via the Baker-Venkataraman reaction [17,18] to produce 1,3-diketones, followed by cyclisation under acidic conditions (Scheme 1). Flavonols **9**–**10** were synthesised from the corresponding 2'-hydroxyacetophenones and benzaldehydes to produce chalcones, followed by cyclisation under the Algar-Flynn-Oyamada [19,20] reaction conditions (Scheme 2). Apigenin (**5**), luteolin (**6**), diosmetin (**7**), fisetin (**8**), kaempferol (**11**), quercetin (**12**) and myricetin (**13**) were obtained from commercial sources.

*Reagents and conditions*: a—pyridine, 110 °C, 2–3 h; b—KOH, pyridine, 60 °C, 4 h, 60%–80% (over 2 steps); c—AcOH, H_2_SO_4_, reflux, 16 h, 35%–55%.

*Reagents and conditions*: a—50% aq. KOH, EtOH, rt, 24 h, 65%–90%; b—30% H_2_O_2_, 20% aq. NaOH, MeOH, 0 °C, 10 h, 50%–75%.

### 2.2. M_1_ mAChR Radioligand Binding Assay

**Figure 2:**
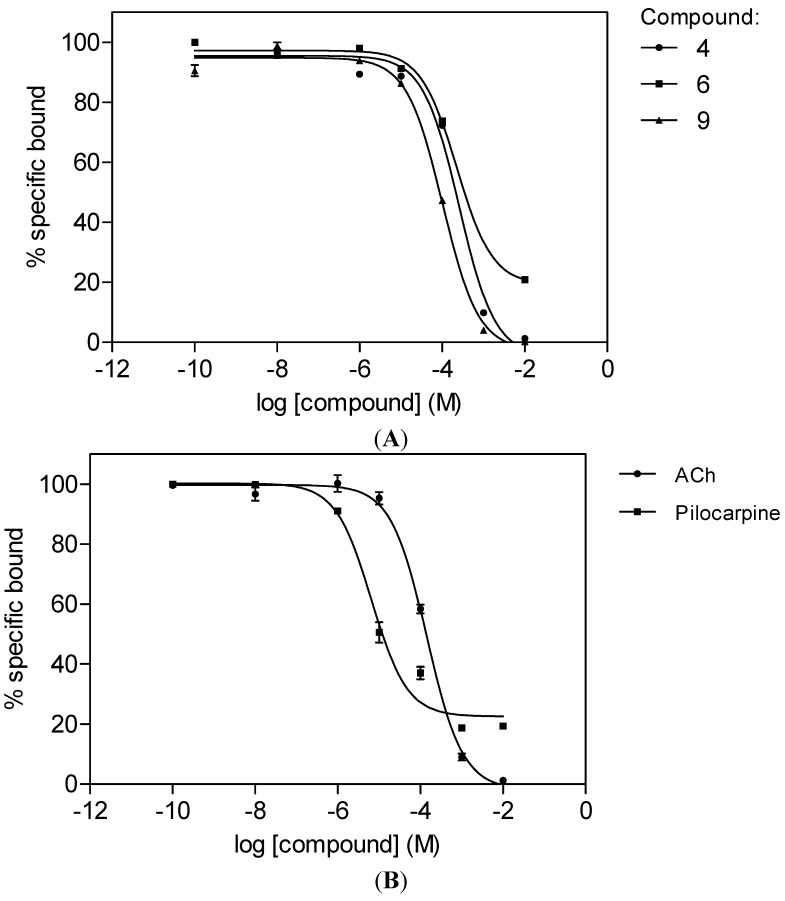
Displacement curves for the binding of [^3^H]N-methylscopolamine to the M_1_ mAChR in the presence of (**A**) 3',4',5',5,6,7-hexamethoxyflavone (**4**), luteolin (**6**) and ombuin (**9**) and (**B**) acetylcholine (ACh) and pilocarpine. Receptor membranes (13 µg protein/well) were incubated with N-methylscopolamine (0.2 nM) in the presence of the compounds at 27 °C for 120 min. Each data point is expressed as a mean ± SEM (*n* = 2).

The compounds were assayed *in vitro* according to the 96-well microplate method previously described [16] for their ability to displace [^3^H]N-methylscopolamine (0.2 nM) using cloned human M_1_ mAChRs (Perkin-Elmer, Boston, MA, USA). Three of the compounds, 3',4',5',5,6,7-hexamethoxyflavone (**4**), luteolin (**6**) and ombuin (**9**), were found to have comparable affinities (*K*_i_ = 40–110 µM) for the M_1_ mAChR with those observed for the standard agonists, acetylcholine (ACh) and pilocarpine (*K*_i_ = 59 and 2.7 µM, respectively) (see Table 1 and Figure 2).

**Table 1:** Substituent patterns and M_1_ mAChR binding data of compounds **1**–**13** and standard agonists.

Cpd	Trivial Name	R_3_	R_5_	R_6_	R_7_	R_3'_	R_4'_	R_5'_	IC_50_ µM	( *K*_i_ µM)
**1**		H	OMe	H	OMe	OMe	OMe	H	>10000	
**2**		H	OMe	H	OMe	OMe	OMe	OMe	>10000	
**3**	Sinensetin	H	OMe	OMe	OMe	OMe	OMe	H	>10000	
**4**		H	OMe	OMe	OMe	OMe	OMe	OMe	257 ± 27	107 ± 11
**5**	Apigenin	H	OH	H	OH	H	OH	H	>10000	
**6**	Luteolin	H	OH	H	OH	OH	OH	H	226 ± 14	94 ± 6
**7**	Diosmetin	H	OH	H	OH	OH	OMe	H	>10000	
**8**	Fisetin	OH	H	H	OH	OH	OH	H	>10000	
**9**	Ombuin	OH	OH	H	OMe	OH	OMe	H	101 ± 6	42 ± 3
**10**		OH	H	OMe	OMe	OMe	OMe	H	>10000	
**11**	Kaempferol	OH	OH	H	OH	H	OH	H	>10000	
**12**	Quercetin	OH	OH	H	OH	OH	OH	H	>10000	
**13**	Myricetin	OH	OH	H	OH	OH	OH	OH	>10000	
	Acetylcholine								142 ± 12	59 ± 5
	Pilocarpine								6.5 ± 0.1	2.7 ± 0.1

### 2.3. Molecular Modelling

To explore the possible binding modes of the compounds and their interactions with the human M_1_ mAChR, molecular modelling studies were carried out. A homology model based on the crystal structure of the rat M_3_ mAChR (PDB code: 4DAJ) [21] was constructed using Prime v3.0 and the orthosteric binding site was refined by induced-fit docking of the endogenous agonist (ACh) using Glide v5.7, as recently described by us elsewhere [22]. Upon docking the three active compounds into the M_1_ mAChR model using AutoDock v4.2 [23], they were all found to bind to the orthosteric site, mainly through non-polar (van der Waals, π-π and hydrophobic) interactions with a number of residues which have been identified by site-directed mutagenesis experiments as being important for the binding of orthosteric ligands [24,25], including Y106, W157, W378, Y381 and Y404.

**Figure 3:**
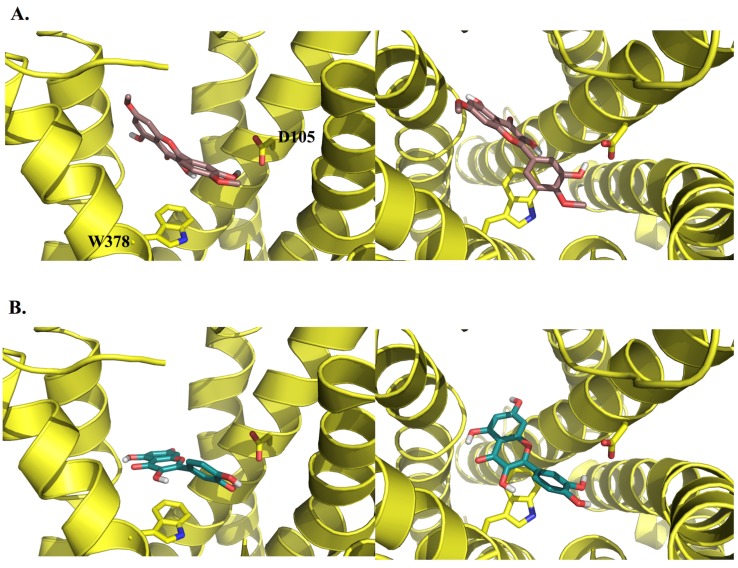
3D representations of the docking poses of (**A**) ombuin (**9**) (brown) and (**B**) quercetin (**12**) (blue-green) in complex with the human M_1_ mAChR model obtained from induced-fit docking using Glide v5.7 and Prime v3.0 (Schrödinger LLC) with side views and top views (from the extracellular surface) depicted in the left hand and right hand panels, respectively. For the purpose of clarity, only D105 and W378 from among the active site residues are depicted in stick representation and some of the transmembrane helices are not shown.

**Figure 4:**
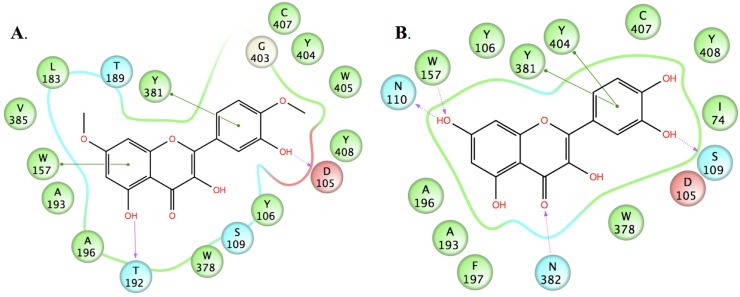
2D representations of the ligand-receptor interactions of (**A**) ombuin (**9**) and (**B**) quercetin (**12**) in complex with the human M_1_ mAChR model obtained from induced-fit docking using Glide v5.7 and Prime v3.0 (Schrödinger LLC), showing pi-pi stacking interactions (green lines), hydrogen bonds involving backbone atoms (solid purple arrows) and hydrogen bonds involving side-chain atoms (dashed purple arrows). Negatively-charged, polar and hydrophobic residues are depicted with red, light blue and green circles, respectively.

In an effort to probe the reasons for the differences in activity between structurally-similar compounds, e.g., ombuin (**9**) *versus* quercetin (**12**) (see Figure 3 and Figure 4), induced-fit docking using Glide and Prime was carried out. The results suggested that the active compounds may be able to make better contacts with key residues, D105, through substituents in ring B, and T192 and N382, through the carbonyl oxygen of the ketone moiety in ring C or the hydroxyl substituent at position 5 in ring A (where present). Alternatively, they may form additional hydrogen bonds to other active site residues.

However, the use of the Prime MM-GBSA module to predict the free energy of binding revealed no significant energy differences for the active compounds compared to the inactive compounds. This may be due to limitations in the docking and scoring functions used, such as not allowing full conformational flexibility in the receptor and excluding the involvement of water molecules in binding.

**Figure 5:**
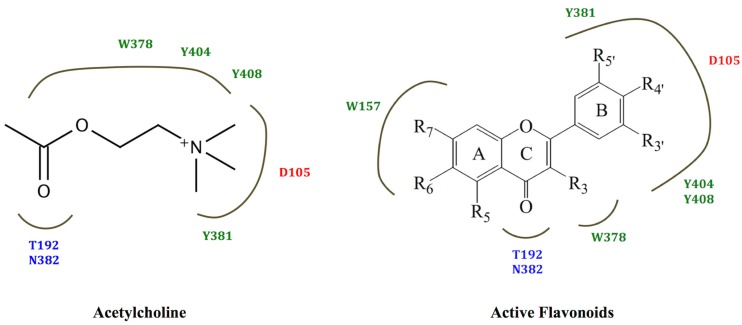
The structural binding analysis map for acetylcholine and a composite map for the active flavonoids, 3',4',5',5,6,7-hexamethoxyflavone (**4**), luteolin (**6**) and ombuin (**9)**, obtained from molecular modelling studies. Negatively-charged, polar and hydrophobic residues are depicted in red, blue and dark green, respectively.

The positively-charged ammonium group that is normally found in mAChR active ligands has been observed to form ionic interactions with the conserved aspartate residue of transmembrane 3 in the two crystal structures of ligands bound to muscarinic receptors that have been solved to date [21,26]. However, the data obtained from this study suggests that the non-polar interactions between a ligand and residues in the hydrophobic cavity of the receptor may be sufficient to compensate for the absence of ionic interactions. The proposal that such non-polar interactions may be of relatively greater importance compared to ionic interactions in the binding of ligands to muscarinic receptors has previously been made from observed differences in affinity within a series of 1,3-oxathiolane compounds [27]. Furthermore, good correlation was observed between the majority of the other key ligand-receptor interactions for acetylcholine and those for the active compounds (see Figure 5). In particular, it appears that the carbonyl oxygen of the ketone moiety in ring C in the flavonoid structures may mimic the carbonyl oxygen in the ester moiety of acetylcholine. However, it should be noted that the effects of the compounds studied as agonists or inverse agonists/antagonists cannot be determined by either radioligand binding or docking studies. These effects would need to be investigated using functional studies.

Interestingly, luteolin (**6**) has previously been observed to exert procognitive effects *in vitro* and *in vivo*. These effects include cholinesterase inhibitory activity [28], protective effects on hydrogen peroxide-induced apoptosis in SH-SY5Y neuroblastoma cells [29], anti-amnesic effects against amyloid *β*_25-35_ peptide-induced toxicity in mice [30] and attenuation of cognitive decline in streptozotocin-induced diabetic rats [31]. In addition, ombuin (**9**) has recently been found to act as a potent inhibitor of DYRK1A/CLK1 kinases, whose increased activity has been linked to neurodegenerative diseases [32]. Hence, the compounds identified in this study have the potential to act as multitarget-directed therapeutic agents, capable of addressing different aspects in the treatment of AD.

**Figure 6:**
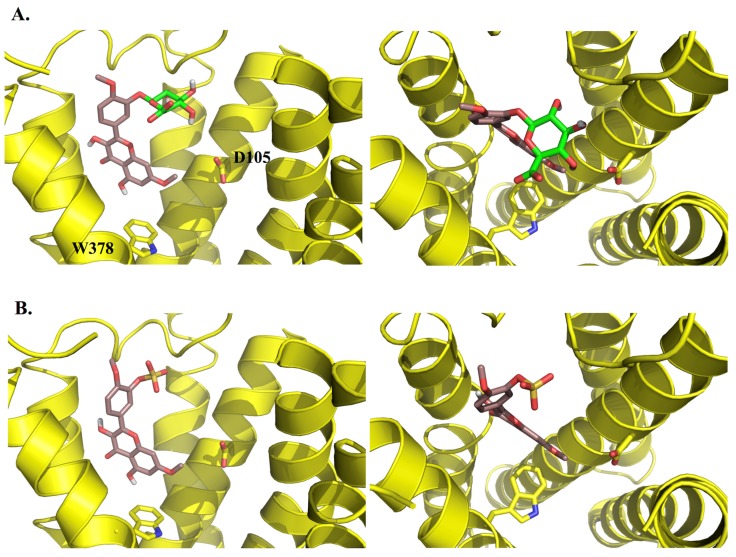
3D representations of the docking poses of (**A**) ombuin-3'-glucuronide and (**B**) ombuin-3'-sulfate in complex with the human M_1_ mAChR model obtained from induced-fit docking using Glide v5.7 and Prime v3.0 (Schrödinger LLC) with side views and top views (from the extracellular surface) depicted in the left hand and right hand panels, respectively. For the purpose of clarity, only D105 and W378 from among the active site residues are depicted in stick representation and some of the transmembrane helices are not shown. The carbon atoms of the glucuronide moiety in ombuin-3'-glucuronide are coloured in light green.

To assess the therapeutic potential of the compounds *in vivo*, bioavailability issues should also be considered. Although orally-administered polyoxygenated flavonoids have been shown to be able to penetrate the blood-brain barrier in rats [33], flavonoids bearing hydroxyl substituents are also known to undergo extensive Phase II metabolism in the course of intestinal absorption. In an effort to predict the effect of such metabolism on the activity of the compounds identified in this study, known metabolites of luteolin (**6**) [34], the 3'-, 4'- and 7-monoglucuronides and the 3'-sulfate, and metabolites of ombuin (**9**) that might be predicted from those that have been observed for quercetin [35,36], the 3'- and 3-monoglucuronides and the 3'-sulfate, were docked into the M_1_ mAChR model using induced-fit docking. They were all found to be able to bind to the active site, but in a different orientation to the aglycones, with the conjugated moiety pointing towards the extracellular surface of the receptor (see Figure 6), suggesting that the activity of the compounds studied may be modified *in vivo*. However, there is also evidence that glucuronoconjugated metabolites of flavonoids are hydrolysed by β-glucuronidase, releasing the parent aglycone [37,38,39,40]. Hence further *in vivo* studies should be carried out to determine the usefulness of compounds which have been found to have good activity *in vitro*.

Lastly, it should be noted that the human dietary intake of flavones and flavonols is only in the range 10–20 mg/day [5], predominantly in the form of quercetin and kaempferol, with luteolin just being found (at levels of 5–20 mg/kg) in specific vegetables, such as celery and capsicum pepper [4]. Furthermore, less commonly available flavonols, such as ombuin, may only be obtained from medicinal plants. Coupled with the bioavailability issues described above, this means that the compounds identified in this study would probably need to be administered in purified form in order to exert significant therapeutic effects *in vivo*.

## 3. Experimental Section

### 3.1. Chemistry

All melting points were taken on a Mel-Temp II melting point instrument. ^1^H and ^13^C-NMR spectra were obtained using Jeol EX 270 (270 MHz) and ECA 400 (400 MHz) NMR spectrometers with TMS as the internal standard. All chemical shifts are reported in ppm. Column chromatography and analytical thin-layer chromatography (TLC) were performed using silica gel 60 (230–400 mesh) and pre-coated aluminum silica gel sheets (Kieselgel 60 F-254) from Merck (Darmstadt, Germany), respectively. The physical and spectroscopic data for flavones **1**–**4** and flavonols **9**–**10** was in agreement with that previously reported for these compounds. All other chemicals used were of analytical grade and were obtained from TransMIT (Marburg, Germany), Sigma-Aldrich (St. Louis, MO, USA) or Merck.

#### 3.1.1. General Procedure for the Synthesis of Flavones

A mixture of the corresponding 2'-hydroxyacetophenone (1 equiv) and benzoyl chloride (1.1 equiv) in pyridine (10 mL/mmol of acetophenone) was heated at 110 °C for 2–3 h. The mixture was then poured into ice and HCl and extracted with ethyl acetate. The extract was washed with water and aqueous sodium carbonate and then dried over sodium sulfate. The solvent was evaporated under reduced pressure to yield the crude phenolic ester. Powdered KOH (4–5 g) was added to a solution of the ester in pyridine (10–15 mL) and the mixture was stirred vigorously at 60 °C for 4 h. The mixture was then poured into ice and HCl, extracted with ethyl acetate and dried over sodium sulphate to give the 1,3-diketone in 60%–80% yield. To a yellow suspension of the 1,3-diketone (2 mmol) in glacial acetic acid (7 mL), concentrated sulfuric acid (a few drops) was added and refluxed overnight. The resulting mixture was cooled and water added until no further white precipitate was formed. The crude solid was filtered, purified using column chromatography and crystallised from methanol to give the flavone in 35%–55% yield.

*3',4',5,7-Tetramethoxyflavone* (**1**): mp 192 °C, lit. 193 °C [41]; ^1^H-NMR (270 MHz, DMSO-*d*_6_): δ 7.60 (d, 8.1 Hz, 1H), 7.49 (s, 1H), 7.07 (d, 8.1 Hz, 1H), 6.86 (s, 1H), 6.70 (s, 1H), 6.46 (s, 1H), 3.86, 3.83, 3.79, 3.78 (s, 3H each); ^13^C-NMR (67.9 MHz, DMSO-*d*_6_): δ 176.48, 164.24, 160.58, 160.48, 159.59, 152.03, 149.35, 123.33, 119.94, 112.08, 108.41, 107.21, 96.66, 93.83, 56.50, 56.27, 56.11.

*3',4',5',5,7-Pentamethoxyflavone* (**2**): mp 195 °C, lit. 198–199 °C [42]; ^1^H-NMR (270 MHz, DMSO-*d*_6_): δ 7.29 (s, 2H), 6.91 (d, 2.2 Hz, 1H), 6.50 (d, 2.2 Hz, 1H), 6.86 (s, 1H), 3.90, 3.89, 3.82, 3.72 (s, 3H each); ^13^C-NMR (67.9 MHz, DMSO-*d*_6_): δ 175.84, 163.78, 160.24, 159.44, 159.23, 153.29, 140.22, 126.25, 108.15, 103.63, 96.33, 93.55, 60.24, 56.22, 56.10, 56.07.

*3',4',5,6,7-Pentamethoxyflavone (sinensetin)* (**3**): mp 176 °C, lit. 174–176 °C [43]; ^1^H-NMR (270 MHz, DMSO-*d*_6_): δ 7.56 (d, 8.0 Hz, 1H), 7.43 (s, 1H), 7.26 (d, 8.0 Hz, 1H), 7.07 (s, 1H), 6.88 (s, 1H), 3.85, 3.78, 3.74, 3.69, 3.66 (s, 3H each); ^13^C-NMR (67.9 MHz, DMSO-*d*_6_): δ 176.42, 160.98, 158.02, 154.47, 152.17, 151.97, 149.45, 140.23, 123.51, 120.10, 112.42, 112.19, 109.51, 106.75, 97.93, 62.44, 61.57, 57.08, 56.42, 53.65.

*3',4',5',5,6,7-Hexamethoxyflavone* (**4**): mp 150 °C, lit. 147–149 °C [44]; ^1^H-NMR (270 MHz, DMSO-*d*_6_): δ 7.32 (s, 2H), 7.13 (s, 1H), 6.94 (s, 1H), 3.91, 3.85 (s, 6H), 3.75, 3.71, 3.68 (s, 3H each); ^13^C-NMR (67.9 MHz, DMSO-*d*_6_): δ 176.34, 160.47, 157.96, 154.34, 153.56, 151.74, 140.59, 140.13, 126.49, 112.25, 107.63, 103.97, 97.91, 62.26, 61.39, 60.58, 56.95, 56.68.

#### 3.1.2. General Procedure for the Synthesis of Flavonols

A mixture of the corresponding 2'-hydroxyacetophenone (1 equiv) and aldehyde (1 equiv) in EtOH (5 mL/1 mmol of acetophenone) was stirred at RT for 30 min. Then a solution of 50% w/w aqueous KOH (1 mL/1 mmol) was added. The reaction mixture was stirred at RT and monitored by TLC until all the aldehyde was consumed. The mixture was then poured into ice-water, acidified with 3N HCl. The crude product was filtered, purified using column chromatography and crystallised from ethanol to give the chalcone in 65%–90% yield. To a solution of the 2'-hydroxychalcone (1 equiv) in methanol (10 mL), 20% w/w aqueous NaOH (2 mL) was added. The solution was cooled to 0 °C and 30% hydrogen peroxide (1 mL) was added dropwise. After 10 h in ice, the product was liberated by ice-cold acetic acid, washed with water, and crystallized from methanol or diluted acetic acid to give the flavonol in 50%–75% yield.

*3ʹ,3,5-Trihydroxy-4ʹ,7-dimethoxyflavone (ombuin)* (**9**): mp 204 °C, lit. 201–203 °C [45]; ^1^H-NMR (400 MHz, DMSO-*d*_6_): δ 12.45 (s, 1H), 9.34 (bs, 1H), 7.63 (s, 1H), 7.64 (d, 8.4 Hz, 1H), 7.03 (d, 8.4 Hz, 1H), 6.64 (d, 2.0 Hz, 1H), 6.29 (d, 2.0 Hz, 1H), 3.81, 3.80 (s, 3H each); ^13^C-NMR (100 MHz, DMSO-*d*_6_): δ 176.45, 165.38, 161.69, 156.69, 149.84, 148.33, 146.79, 136.65, 123.90, 120.56, 116.07, 115.06, 104.49, 98.73, 93.93, 56.84, 55.43.

*3-Hydroxy-3ʹ,4ʹ,6,7-tetramethoxyflavone* (**10**): mp 225–226 °C, lit. 226 °C [46]; ^1^H-NMR (400 MHz, CDCl_3_): δ 7.76 (d, 9.0 Hz, 1H), 7.73 (s, 1H), 7.43 (s, 1H), 6.92 (d, 9.0 Hz, 1H), 6.89 (s, 1H), 3.95, 3.93, 3.92, 3.89 (s, 3H each); ^13^C-NMR (100 MHz, CDCl_3_): δ 172.03, 154.60, 151.64, 150.37, 148.77, 147.40, 144.35, 137.30, 123.91, 120.95, 113.95, 110.84, 110.41, 103.51, 99.54, 56.46, 56.31, 56.00, 55.91.

### 3.2. M_1_ mAChR Radioligand Binding Assay

Radioligand binding assays were carried out as previously described [16], but using M_1_ mAChR membranes from stably transfected CHO-K1 cell lines (Perkin Elmer, Waltham, MA, USA). Around 13 µg/well of M_1_ mAChR membrane suspension in 170 µL/well PBS (pH 7.4) was added to a mixture of test compound in 0.25% v/v of DMSO (10 µL/well; 10^−2^ M–10^−8^ M, final concentration), [3H]N-methylscopolamine (Amersham Pharmacia Biotech., Little Chalfont, Buckinghamshire, UK) (10 µL/well; 0.2 nM, final concentration), guanosine triphosphate (10 µL/well; 0.1 mM, final concentration). After 120 min of incubation at 27 °C, the reaction mixture was filtered with a GF/C filter plate on a UniFilter cell harvester (Perkin-Elmer) and washed with 200 µL/well of ice cold 50 mM Tris-HCl buffer (pH 7.4) (nine times). The GF/C filter plate was then air-dried for 24 h before the application of Bottom Seal and addition of MicroScint-O scintillation cocktail (25 µL/well) (Perkin-Elmer)*.* The top of the plate was then sealed with TopSeal A. After 1 h the plate was counted for 1 min/well with a TopCount NXT microplate scintillation counter (Perkin-Elmer). Non-specific binding was determined by adding atropine (10 µL/well; 5 µM, final concentration) to the reaction mixture. The percentage of specific binding of radioligand was calculated using the standard data reduction algorithm:

([B − NSB]/[TB − NSB]) × 100

where B is the binding in the presence of a test compound, NSB is the non-specific binding in the presence of excess reference ligand and TB is the total binding.

Data analysis was performed with PRISM^®^ v5.0 (GraphPad, San Diego, CA, USA). Values of *K*_d_ (the equilibrium dissociation constant) for the radioligand, B_max_ (maximum number of binding sites for the radioligand) and IC_50_ (the half maximal inhibitory concentration) for the test compounds were obtained as the best-fit values for the data from non-linear regression analysis using the least-squares method. Inhibition constant (*K*_i_) values were calculated using the Cheng-Prusoff Equation [47]:

*K*_i_ = IC_50_/(1 + [L]/*K*_d_)

where [L] is the radioligand concentration that was used. Values are expressed as mean (*n* = 2) ± SEM.

### 3.3. Molecular Modelling

#### 3.3.1. Homology Modelling of the M_1_ mAChR

The sequence of the human M_1_ mAChR (accession number: P11229) was retrieved from the Swiss-Prot sequence database [48]. Guided alignment of the human M_1_ receptor sequence and that obtained from the structure of the rat M_3_ mAChR complexed with an inverse agonist (PDB code: 4DAJ) [21] was carried out using Expresso [49]. A homology model of the M_1_ receptor was then constructed using Prime v3.0 (Schrödinger LLC, New York, NY, USA), as previously described [22]. Briefly, ECLs and ICLs connecting the TM domains were modelled according to the template structure except ICL3, which was excluded from the modelling. Binding site refinement was carried out by induced-fit docking (IFD) of the endogenous muscarinic receptor agonist (ACh) using Glide v5.7 (Schrödinger LLC) and side chain refinement using Prime v3.0. The IFD protocol began with a constrained minimization of the receptor structure, followed by initial Glide docking of ACh using a softened potential, to allow more poses of the ligand to be generated and collected. One round of Prime side-chain prediction was then carried out for the residues found within 5 Å of the ligand, followed by minimization of each ligand-receptor complex. Finally, the ligand was re-docked into the induced-fit receptor structure, without softened potential and the binding energy or IFD score was obtained. After multiple iterations of docking and binding site refinement, an initial set of models was chosen on the basis of showing the expected ligand-receptor interactions. The resulting ligand-receptor conformations were inspected visually to ensure that the quaternary amine group of the docked ACh was directed towards D105 and the side chains of the orthosteric site residues were facing inwards towards the inner channel of the TM region. The best model was then selected on the basis of their ability to differentiate active compounds from decoys.

#### 3.3.2. Docking Studies

Initial docking studies were performed with AutoDock 4.2, using a Lamarckian genetic algorithm [50] with a flexible ligand and a rigid receptor, a population size of 300, a maximum of 250,000 generations and 2,500,000 evaluations for 100 GA runs. AutoDockTools4 [23] was used to prepare the input files and docking grids and the grid box was set to cover the TM domain. The root mean square deviation (RMSD) tolerance was set to 2.0 Å for the clustering of docked results. Ligand-receptor interactions were viewed using Discovery Studio Visualizer v3.1 (Accelrys, San Diego, CA, USA).

Induced-fit docking was performed using the same parameters and protocol as the binding site refinement of the model (see Section 3.3.1). The best docked conformation were selected based on the induced-fit docking score, GScore, and the observed interactions with the active site residues. Docking poses and ligand receptor interactions were viewed using PyMOL and Maestro (Schrödinger LLC), respectively. The obtained poses were re-scored using the Prime MM-GBSA module to predict the free energy of binding (∆*G*_bind_) with the following equation:

∆*G*_bind_ = *G*_complex_ − (*G*_protein_ + *G*_ligand_)

where *G*_complex_, *G*_protein_ and *G*_ligand_ are the optimized free energies of binding for the protein-ligand complex, the free protein and free ligand, respectively [51,52]. Each energy term was calculated by a combination of molecular mechanics energy, implicit solvation energy and surface area energy. Residues in the binding pocket of the receptor were treated as flexible.

## 4. Conclusions

In this study, a series of natural and synthetic flavones and flavonols was assayed *in vitro* for their ability to inhibit radioligand binding at human cloned M_1_ muscarinic acetylcholine receptors. Several compounds were found to possess competitive binding affinity (*K*_i_ = 40–110 µM), comparable to that of acetylcholine (*K*_i_ = 59 µM). Molecular modelling studies suggested that the compounds bind to the orthosteric site of the receptor, mainly through non-polar interactions. Although no significant energy differences were observed for the binding of the active compounds compared to the inactive compounds, this may be due to limitations in the docking and scoring functions used. Further studies are required in order to determine whether the compounds act as agonists or inverse agonists/antagonists, possess any sub-type selectivity and have sufficient bioavailability *in vivo*. The results from such studies will be able to give more of an indication of the potential of flavonoid compounds for the treatment of AD.

## References

[1] Fisher A. (2008). Cholinergic treatments with emphasis on M1 muscarinic agonists as potential disease-modifying agents for Alzheimer’s disease. Neurotherapeutics.

[2] Langmead C.J., Watson J., Reavill C. (2008). Muscarinic acetylcholine receptors as CNS drug targets. Pharmacol. Ther..

[3] Davis A.A., Fritz J.J., Wess J., Lah J.J., Levey A.I. (2010). Deletion of M1 muscarinic acetylcholine receptors increases amyloid pathology *in vitro* and *in vivo*. J. Neurosci..

[4] Manach C., Scalbert A., Morand C., Remesy C., Jimenez L. (2004). Polyphenols: Food sources and bioavailability. Am. J. Clin. Nutr..

[5] Chun O.K., Chung S.J., Song W.O. (2007). Estimated dietary flavonoid intake and major food sources of U.S. adults. J. Nutr..

[6] Vauzour D., Vafeiadou K., Rodriguez-Mateos A., Rendeiro C., Spencer J.P.E. (2008). The neuroprotective potential of flavonoids: A multiplicity of effects. Genes Nutr..

[7] Hwang S.-L., Shih P.-H., Yen G.-C. (2012). Neuroprotective effects of citrus flavonoids. J. Agric. Food Chem..

[8] Jäger A.K., Saaby L. (2011). Flavonoids and the CNS. Molecules.

[9] Williams R.J., Spencer J.P.E. (2012). Flavonoids, cognition, and dementia: Actions, mechanisms, and potential therapeutic utility for Alzheimer disease. Free Radic. Biol. Med..

[10] Commenges D., Scotet V., Renaud S., Jacqmin-Gadda H., Barberger-Gateau P., Dartigues J.-F. (2000). Intake of flavonoids and risk of dementia. Eur. J. Epidemiol..

[11] Letenneur L., Proust-Lima C., le Gouge A., Dartigues J.F., Barberger-Gateau P. (2007). Flavonoid intake and cognitive decline over a 10-year period. Am. J. Epidemiol..

[12] Banister J., Whittaker V.P. (1951). Pharmacological activity of the carbon analogue of acetylcholine. Nature.

[13] Barlow R.B., Tubby J.H. (1974). Actions of some esters of 3,3-dimethylbutan-1-ol (the carbon analogue of choline) on the guinea-pig ileum. Br. J. Pharmacol..

[14] Barlow R.B., Bond S., Holdup D.W., Howard J.A.K., McQueeen D.S., Paterson A., Veale M.A. (1992). The contribution of charge to affinity at functional (M3) muscarinic receptors in guinea-pig ileum assessed from the effects of the carbon analogue of 4-DAMP methiodide. Br. J. Pharmacol..

[15] Waelbroeck M., Hou X., Wehrle J., Mutschler E., van Tilburg E., Menge W., Timmerman H., Lambrecht G. (1996). Stereoselective interaction of uncharged esters at four muscarinic receptor subtypes muscarinic receptor subtypes. Eur. J. Pharmacol..

[16] Chung L.Y., Yap K.F., Goh S.H., Mustafa M.R., Imiyabir Z. (2008). Muscarinic receptor binding activity of polyoxygenated flavones from *Melicope subunifoliolata*. Phytochemistry.

[17] Baker W. (1933). Molecular rearrangement of some o-acyloxyacetophenones and the mechanism of the production of 3-acylchromones. J. Chem. Soc..

[18] Mahal H.S., Venkataraman K. (1933). A synthesis of flavones at room temperature. Curr. Sci..

[19] Algar J., Flynn J.P. (1934). New synthesis of flavonols. Proc. R. Irish Acad..

[20] Oyamada B. (1935). A new general method for the synthesis of the derivatives of flavonol. Bull. Chem. Soc. Jpn..

[21] Kruse A.C., Hu J., Pan A.C., Arlow D.H., Rosenbaum D.M., Rosemond E., Green H.F., Liu T., Chae P.S., Dror R.O. (2012). Structure and dynamics of the M3 muscarinic acetylcholine receptor. Nature.

[22] Chin S.P., Buckle M.J.C., Chalmers D.K., Yuriev E, Doughty S.W. (2014). Toward activated homology models of the human M1 muscarinic acetylcholine receptor. J. Mol. Graph. Model..

[23] Morris G.M., Huey R., Lindstrom W., Sanner M.F., Belew R.K., Goodsell D.S., Olson A.J. (2009). AutoDock4 and AutoDockTools4: Automated docking with selective receptor flexibility. J. Comput. Chem..

[24] Hulme E.C., Lu Z.L., Bee M.S. (2003). Scanning mutagenesis studies of the M1 muscarinic acetylcholine receptor. Recept. Channels.

[25] Goodwin J.A., Hulme E.C., Langmead C.J., Tehan B.G. (2007). Roof and floor of the muscarinic binding pocket: Variations in the binding modes of orthosteric ligands. Mol. Pharmacol..

[26] Haga K., Kruse A.C., Asada H., Yurugi-Kobayashi T., Shiroishi M., Zhang C., Weis W.I., Okada T., Kobilka B.K., Haga T. (2012). Structure of the human M2 muscarinic acetylcholine receptor bound to an antagonist. Nature.

[27] Gualtieri F., Romanelli M.N., Teodori E. (1990). Eudismic analysis of a series of muscarinic ligands carrying a 1,3-oxathiolane nucleus. Chirality.

[28] Conforti F., Rigano D., Formisano C., Bruno M., Loizzo M.R., Menichini F., Senatore F. (2010). Metabolic profile and and *in vitro* activities of *Phagnalon saxatile* (L.) Cass. relevant to treatment of Alzheimer’s disease. J. Enzym. Inhib. Med. Chem..

[29] Kang S.S., Lee J.Y., Choi Y.K., Kim G.S., Han B.H. (2004). Neuroprotective effects of flavones on hydrogen peroxide-induced apoptosis in SH-SY5Y neuroblostoma cells. Bioorg. Med. Chem. Lett..

[30] Liu R., Gao M., Qiang G.F., Zhang T.T., Lan X., Ying J., Du G.H. (2009). The anti-amnesic effects of luteolin against amyloid *β*(25-35) peptide-induced toxicity in mice involve the protection of neurovascular unit. Neuroscience.

[31] Liu Y., Tian X., Gou L., Sun L., Ling X., Yin X. (2013). Luteolin attenuates diabetes-associated cognitive decline in rats. Brain Res. Bull..

[32] Grabher P., Durieu E., Kouloura E., Halabalaki M., Skaltsounis L.A., Meijer L., Hamburger M., Potterat O. (2012). Library-based discovery of DYRK1A/CLK1 inhibitors from natural product extracts. Planta Med..

[33] Rangel-Ordóñez L., Nöldner M., Schubert-Zsilavecz M., Wurglics M. (2010). Plasma levels and distribution of flavonoids in rat brain after single and repeated doses of standardized ginkgo biloba extract EGb 761^®^. Planta Med..

[34] Boersma M.G., van der Woude H., Bogaards J., Boeren S., Vervoort J., Cnubben N.H., van Iersel M.L., van Bladeren P.J., Rietjens I.M. (2002). Regioselectivity of phase II metabolism of luteolin and quercetin by UDP-glucuronosyl transferases. Chem. Res. Toxicol..

[35] Day A.J., Bao Y., Morgan M.R., Williamson G. (2000). Conjugation position of quercetin glucuronides and effect on biological activity. Free Radic. Biol. Med..

[36] Day A.J., Mellon F., Barron D., Sarrazin G., Morgan M.R., Williamson G. (2001). Human metabolism of dietary flavonoids: Identification of plasma metabolites of quercetin. Free Radic. Res..

[37] O’Leary K.A., Day A.J., Needs P.W., Sly W.S., O’Brien N.M., Williamson G. (2001). Flavonoid glucuronides are substrates for human liver β-glucuronidase. FEBS Lett..

[38] Shimoi K., Saka N., Nozawa R., Sato M., Amano I., Nakayama T., Kinae N. (2001). Deglucuronidation of a flavonoid, luteolin monoglucuronide, during inflammation. Drug Metab. Dispos..

[39] Bartholomé R., Haenen G., Hollman C.H., Bast A., Dagnelie P.C., Roos D., Keijer J., Kroon P.A., Needs P.W., Arts I.C. (2010). Deconjugation kinetics of glucuronidated phase II flavonoid metabolites by *β*-glucuronidase from neutrophils. Drug Metab. Pharmacokinet..

[40] Menendez C., Dueñas M., Galindo P., González-Manzano S., Jimenez R., Moreno L., Zarzuelo M.J., Rodríguez-Gómez I., Duarte J., Santos-Buelga C. (2011). Vascular deconjugation of quercetin glucuronide: The flavonoid paradox revealed?. Mol. Nutr. Food Res..

[41] Sutthanut K., Sripanidkulchai B., Yenjai C., Jay M. (2007). Simultaneous identification and quantitation of 11 flavonoid constituents in *Kaempferia parviflora* by gas chromatography. J. Chromatogr. A.

[42] Mateeva N.N., Kode R.N., Redda K.K. (2002). Synthesis of novel flavonoid derivatives as potential HIV-integrase inhibitors. J. Heterocycl. Chem..

[43] Chu H.W., Wu H.T., Lee Y.J. (2004). Regioselective hydroxylation of 2-hydroxychalcones by dimethyldioxirane towards polymethoxylated flavonoids. Tetrahedron.

[44] Kong C., Liang W., Hu F., Xu X., Wang P., Jiang Y., Xing B. (2004). Allelochemicals and their transformations in the *Ageratum conyzoides* intercropped citrus orchard soils. Plant Soil.

[45] Huong D.T., Luong D.V., Thao T.T., Sung T.V. (2005). A new flavone and cytotoxic activity of flavonoid constituents isolated from Miliusa balansae (Annonaceae). Pharmazie.

[46] King F.E., King T.J., Neill K.G. (1953). The chemistry of extractives from hardwoods. Part XI. The isolation of a diterpene ester (methyl vinhaticoate), and of 6:7:3':4'-tetrahydroxyflavanone (plathymenin), and 2:4:5:3':4'-pentahydroxychalkone (neoplathymenin), from Plathymenia reticulate. J. Chem. Soc..

[47] Cheng Y.C., Prusoff W.H. (1973). Relationship between the inhibition constant (K1) and the concentration of inhibitor which causes 50 per cent inhibition (I50) of an enzymatic reaction. Biochem. Pharmacol..

[48] Boeckmann B., Bairoch A., Apweiler R., Blatter M.C., Estreicher A., Gasteiger E., Martin M.J., Michoud K., O’Donovan C., Phan I. (2003). The Swiss-Prot protein knowledgebase and its supplement TrEMBL in 2003. Nucleic Acids Res..

[49] Armougom F., Moretti S., Poirot O., Audic S., Dumas P., Schaeli B., Keduas V., Notredame C. (2006). Expresso: Automatic incorporation of structural information in multiple sequence alignments using 3D-Coffee. Nucleic Acids Res..

[50] Morris G.M., Goodsell D.S., Halliday R.S., Huey R., Hart W.E., Belew R.K., Olson A.J. (1998). Automated docking using a Lamarckian genetic algorithm and an empirical binding free energy function. J. Comput. Chem..

[51] Jacobson M.P., Friesner R.A., Xiang Z., Honig B. (2002). On the role of the crystal environment in determining protein side-chain conformations. J. Mol. Biol..

[52] Jacobson M.P., Pincus D.L., Rapp C.S., Day T.J.F., Honig B., Shaw D.E., Friesner R.A. (2004). A hierarchical approach to all-atom protein loop prediction. Proteins.

